# Factors Affecting Patients’ Use of Telehealth Services: Cross-Sectional Survey Study

**DOI:** 10.2196/63295

**Published:** 2025-07-10

**Authors:** Jiajia Qu

**Affiliations:** 1 Computer Information Systems and Business Analytics, James Madison University, 800 South Main Street, Harrisonburg, VA, 22807, United States, 1 7164586270

**Keywords:** HINTS dataset, telehealth utilization, confidence in health information seeking, patient-centered communication, health literacy, trust, social determinants of health, health self-efficacy, structural equation modeling

## Abstract

**Background:**

The increased integration of telehealth services into health care systems, especially during the COVID-19 pandemic, transformed patient-provider interactions. Despite numerous benefits that promote health equity and resource allocation, patients’ acceptance and use of telehealth have declined post pandemic. To enhance health care delivery and patient satisfaction, we study the factors of this decline from the perspective of patient characteristics that influence the adoption and use of telehealth services.

**Objective:**

This study examines the direct impact of patient trust, social determinants of health, and health self-efficacy on telehealth usage, the indirect effect of confidence in health information seeking, patient-centered communication, and health literacy barriers on telehealth usage through trust.

**Methods:**

This paper uses secondary data from cycle 6 of the Health Information National Trends Survey, a nationally representative dataset collected by the National Cancer Institute. This dataset used a mixed-mode experimental design, with data collected between March and November 2022. The survey included 2 experimental conditions: concurrent (web and paper surveys offered simultaneously) and sequential (web survey offered first, followed by paper). A total of 6252 respondents participated, with a household response rate of 28.1% (6252/22,471). Respondents were randomly assigned to 1 of 3 web-based survey groups to address data quality issues such as speeding and straight lining. We use structural equation modeling to test our research questions, evaluating both direct and indirect pathways influencing telehealth usage. Common method bias is addressed through Harman’s single-factor test, and robustness checks ensure the validity and reliability of our results.

**Results:**

Out of 5554 participants who had at least 1 doctor visit within the past 12 months, 44.89% used telehealth services in the past year. Trust has an inverted U-shaped relationship with confidence in health information seeking (β=−.031; *P*=.002); we find trust positively influenced by patient-centered communication (β=.156; *P<*.001) and negatively affected by health literacy barriers (β=−.063; *P<*.001). Trust enhances telehealth usage (β=.025; *P<*.001), with social determinants of health exerting a positive impact (β=.105; *P<*.001) and health self-efficacy having a negative impact (β=−.019; *P*=.007).

**Conclusions:**

This study finds that trust, social determinants of health, and health self-efficacy directly impact telehealth usage. Additionally, telehealth usage is indirectly influenced by patient characteristics, such as confidence in health information seeking and health literacy barriers, as well as by a patient-centered communication environment. The findings emphasize the need for targeted interventions to improve patient health literacy and engagement, thereby promoting the telehealth services usage.

## Introduction

### Background

Telehealth refers to the remote delivery of health care services through technology, including video consultations, phone calls, and asynchronous communication, representing a paradigm shift in health care delivery. As a technological innovation in the health care delivery model, telehealth reshapes patient access and medical care receipt patterns [1,2]. The global COVID-19 pandemic accelerated the integration of telehealth into health care systems [3]. According to statistics, the number of telehealth visits increased by 154% in the first quarter of 2020 compared with the same period in 2019 [4], highlighting the rapid expansion of telehealth usage in response to the pandemic. This surge underscores telehealth’s crucial role in maintaining health care access during public health emergencies. However, in the postpandemic era, the challenge remains of sustainably scaling telehealth innovations and optimizing patient services within the health care service model. Despite the continued advocacy for telehealth services by health care providers and insurance companies due to the great convenience and optimized resource allocation, telehealth usage has declined by 45.8% since 2022 [5].

As our study seeks to understand how patient characteristics impact telehealth usage, we are grounded in 3 key theoretical frameworks: the Technology Acceptance Model (TAM), the Health Belief Model (HBM), and the Social Ecological Model (SEM). The TAM posits that perspective usefulness and trust significantly influence technology adoption behavior [6]. In telehealth, trust in health care providers and digital platforms determines patients’ willingness to adopt virtual care, making trust a critical factor in telehealth usage [7]. The HBM highlights the role of health self-efficacy (HSE)—an individual’s belief in their ability to manage and access health—in health-related decisions, including during the engagement with health care services and whether such engagement builds trust [7,8]. Finally, the SEM emphasizes the impact of social determinants of health (SDOH) on health care access, suggesting that structural barriers such as food, housing, and transportation insecurity can either facilitate or inhibit telehealth adoption [9]. These models collectively provide a foundation for this research. Given the central role of trust in telehealth usage, this study examines how patient-centered communication (PCC), health literacy barriers, and confidence in health information seeking influence trust, and in turn, how trust, SDOH, and HSE jointly impact telehealth usage.

To summarize, this study addresses the following research questions: (1) How do trust, SDOH—specifically food, housing, and transportation insecurity—and patient HSE influence telehealth usage? (2) How does trust mediate the relationship between patients’ confidence in health information seeking, PCC, and health literacy barriers in shaping telehealth usage?

This study contributes to the literature by integrating perspectives from the TAM, HBM, and SEM to examine how trust, SDOH, and HSE jointly influence telehealth usage. Unlike previous studies that focus on isolated factors, our research provides a holistic framework that captures the interplay between patient trust, socioeconomic barriers, and individual capabilities in shaping telehealth adoption. By doing so, we offer new insights into reducing disparities in telehealth usage and optimizing patient-centered digital health care delivery.

### Literature Review

#### Telehealth Usage

Previous research has explored various factors influencing telehealth usage, including technology acceptance [10,11], provider recommendations during services [12,13], and insurance coverage [14]. Additionally, patient characteristics, including geographic location [3], health literacy barriers [15], and patient trust [16], have also been shown to influence telehealth usage. Using the same Health Information National Trends Survey (HINTS) dataset, Mojtahedi et al [17] analyzed telehealth usage among informal caregivers and found that telehealth usage shows significant inequity through technical problems and census regions. However, these studies have largely focused on isolated factors, with limited integration of the patient-centered perspective. Our study addresses these gaps by examining how trust, SDOH, and HSE jointly influence telehealth usage, as well as how trust mediates the impact of PCC, health literacy barriers, and confidence in health information seeking.

#### Trust

Telehealth, as a web-based health care platform, fundamentally relies on the exchange of health knowledge and information between health care professionals and patients via the web. Trust in the context of telehealth refers to patients’ confidence in the competence, benevolence, and integrity of health care providers and digital health platforms, which is a significant challenge in the virtual setting [16,18].

According to the TAM, trust significantly influences patients’ perceptions of telehealth’s usefulness and ease of use, thereby shaping their adoption behavior [6,7]. PCC, which emphasizes shared decision-making and responsiveness to patient concerns, fosters stronger provider-patient relationships and enhances trust in health recommendations [19,20]. Furthermore, confidence in health information seeking, which represents a patient’s ability to independently find and interpret medical information, plays a critical role in establishing trust in both digital and traditional health care settings [21]. Patients with low confidence in their ability to evaluate web-based health information may be more skeptical of telehealth platforms and digital consultations. Similarly, health literacy barriers—such as difficulties in understanding medical information and navigating digital health resources—can limit a patient’s ability to confidently engage with health care providers, thereby weakening trust [22]. Our study explores how to enhance trust to optimize telehealth services by examining the impact of patient confidence in health information seeking, PCC, and health literacy barriers on trust building. By addressing these factors, we hope to provide insights that can improve telehealth adoption and create a more supportive environment for patients.

#### Confidence in Health Information Seeking

Confidence in health information seeking reflects an individual’s self-reported belief in their ability to find and evaluate health-related information on the web, which can influence their trust in health care providers and their willingness to use telehealth services [23]. Research indicates that individuals with high confidence in health information seeking are more likely to actively seek health information and engage with digital health platforms, resulting in greater trust and engagement during health care services [24]. However, excessive confidence may lead to overreliance on unverified web-based sources, potentially undermining trust in health care providers [25]. Our study examines the role of confidence in health information seeking in shaping trust and telehealth usage, building on prior research that has used similar measures to assess this construct [23].

#### Patient-Centered Communication

PCC involves actively listening to patients’ concerns, preferences, and needs, fostering stronger provider-patient relationships and enhancing trust [19,20,26]. Studies have demonstrated that PCC improves service satisfaction, treatment adherence, and health outcomes [27]. Additionally, a study reported that PCC practices led to higher patient satisfaction and adherence to treatment plans [28,29], which are essential for effective telehealth usage. Therefore, understanding the role of PCC in fostering trust and its subsequent influence on telehealth usage is vital for promoting a supportive and effective telehealth environment.

#### Health Literacy Barriers

Health literacy barriers, such as difficulties in understanding medical information, can limit patients’ ability to confidently engage with health care providers, thereby weakening trust [22]. Previous research highlights the importance of health literacy, which empowers patients to actively engage during the service process and thus facilitate health care access, effective communication, and chronic disease management [15,30]. Given the user-generated nature of content on online platforms during telehealth services, patients must use their health literacy skills to differentiate truthful content and build trust [24]. Studies show that patients with lower health literacy barriers have higher potential to engage in health care services, trust health recommendations, and achieve better health care outcomes [30], while high health literacy barriers prohibit patients from obtaining effective and high-quality care, especially for disadvantaged groups [31]. Additionally, patients with health literacy barriers are less likely to build the trust in a web-based environment needed to use medical websites for health information [24]. Building on existing literature, we aim to understand how health literacy barriers impact trust and, in turn, influence telehealth usage.

#### Social Determinants of Health

The Healthy People 2020 report defines SDOH as environmental conditions influencing various aspects of people’s lives, including health outcomes and risks [32]. SDOH encompass various factors that impact unequal health outcomes and access to care. Previous studies have highlighted the challenges and insecurities associated with SDOH in promoting telehealth, such as unrepresentative cultural backgrounds [33]. These factors suggest that individuals facing constraints in SDOH are less likely to use telehealth services. However, telehealth offers potential solutions to SDOH barriers related to transportation, geography, and flexibility when accessing health care services [34]. Given the conflicting roles of SDOH, our study builds on existing research by examining whether SDOH, specifically food, housing, and transportation insecurity, would promote the use of telehealth services.

#### Health Self-Efficacy

HSE refers to an individual’s confidence in effectively managing their health [30]. Existing literature highlights its significance in determining health care outcomes, as patients with higher HSE are more likely to engage in health-promoting behaviors and navigate health care systems adeptly [30,35]. Within the current research landscape, 2 perspectives have emerged regarding the impact of HSE on telehealth usage. One perspective suggests that individuals with greater confidence in managing their health, indicative of higher HSE levels, exhibit increased engagement in telehealth services and report greater satisfaction [35]. Conversely, some studies propose that individuals with higher HSE may perceive less need for telehealth services, feeling capable of managing their health independently or preferring active engagement in traditional health care service processes [36]. Additionally, individuals with higher HSE may expect real-time communication and interactions, potentially leading to skepticism regarding the use of telehealth and reluctance to adopt it. Given the uncertain role of HSE in telehealth usage, our paper investigates the impact of HSE on telehealth usage, given the synergistic effects of SDOH and trust.

Overall, this study extends prior research by integrating perspectives from the TAM, HBM, and SEM to provide a more holistic understanding of telehealth usage. Unlike previous studies that primarily focus on technological or provider-side factors, our research highlights how patient trust, HSE, and SDOH jointly influence digital health care adoption, offering new insights into reducing telehealth disparities.

### Hypothesis Development

**Figure 1.**
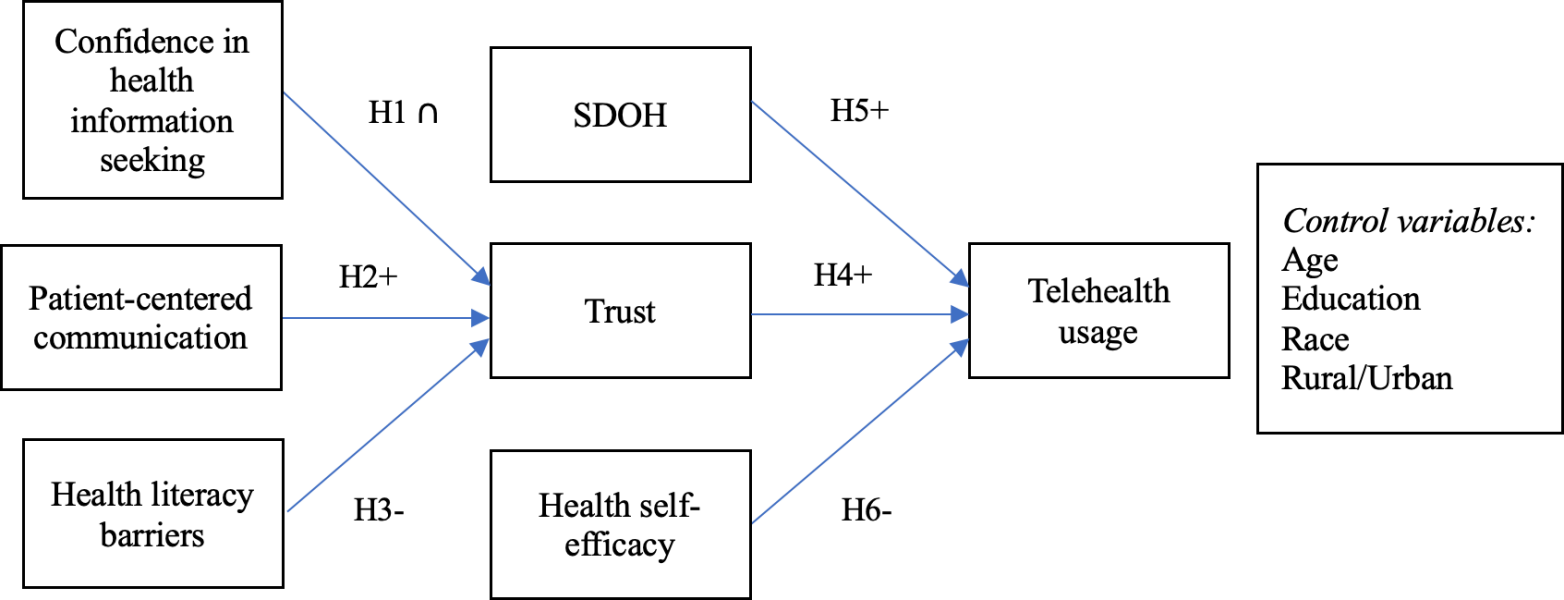
Research model. SDOH: social determinants of health.

Building on prior work, this study examines how trust, SDOH, and HSE influence telehealth usage. Additionally, we investigate how patient characteristics impact trust through 3 key factors: confidence in health information seeking, PCC, and health literacy barriers. These interrelationships are illustrated in Figure 1, providing a comprehensive framework for our research questions.

#### Confidence in Health Information Seeking and Trust

When patients’ confidence in finding reliable health information increases, they tend to view doctors and other medical professionals as trusted sources and rely more on them for accurate health information [37]. However, as patients become more proficient in seeking health-related information on the web, they gain a sense of empowerment, and their trust in health care providers and professionals may diminish [25]. For instance, when patients’ confidence in health information seeking reaches a high level, they may become more independent in their thinking and reduce their trust in health care providers. Based on this discussion, we propose our first hypothesis as follows:

- Hypothesis 1: Patients’ confidence in health information seeking has an inverted U-shaped association with trust, where the effect of confidence in health information seeking is positive at low levels but becomes negative at high levels.

#### PCC and Trust

PCC, marked by empathy, active listening, and shared decision-making, plays a significant role in shaping patients’ perceptions of benefits and develops higher trust in their health care providers [19,20,29]. When health care providers offer a PCC environment, they establish a supportive and trusting relationship with patients, which enhances communication effectiveness and patient satisfaction, as patients feel more confident in the competence and compassion of their providers [20]. Based on this argument, we propose the following hypothesis:

- Hypothesis 2: PCC is positively associated with trust.

#### Health Literacy Barriers

Patients with limited health literacy or facing health literacy barriers need help understanding health care information, engaging with the health care system, and interacting with health care providers [15,31]. This can lower their expected satisfaction with the overall health service process and undermine trust toward providers [24]. The HBM also suggests that individuals’ belief about their susceptibility to health conditions influences their trust in health care providers and adherence to care [27,38], indicating that lower health literacy harms trust. In this case, we propose the following hypothesis:

- Hypothesis 3: Health literacy barriers are negatively associated with trust.

#### Trust and Telehealth Usage

The TAM posits that individuals’ perception of the usefulness and ease of use of technology and their trust in the system influence their acceptance and adoption of technology-based innovation, such as telehealth [6,7]. Also, previous literature highlights the importance of building trust between health care providers and government agencies regarding telehealth adoption. It suggests that trust is the basis for any new health care delivery model [22], and that it is critical to understand the dynamics of trust in patients’ attitudes toward telehealth adoption. Based on this discussion, we propose the following hypothesis:

- Hypothesis 4: Trust is positively associated with telehealth usage.

#### SDOH and Telehealth Usage

Patients’ social environment, including household income and reliable transportation constraints, can be considered enabling factors influencing telehealth usage. Telehealth offers more affordable options that may encourage low-income patients to choose telehealth services. Accordingly, we propose the following hypothesis:

- Hypothesis 5: SDOH (food, housing, and transportation insecurity) are positively associated with telehealth usage.

#### HSE and Telehealth Usage

Following previous research, this study defines HSE as an individual’s belief that they can perform a specific act conducive to their health without being restricted by telehealth barriers [38]. As discussed in the literature section, HSE plays a contradictory role in telehealth usage. As individuals with HSE anticipate more positive outcomes in building and maintaining supportive relationships, HSE may discourage the remote care delivery model. The HBM also offers insights into the relationship between HSE and telehealth services usage [38]. According to the HBM, patients’ perceived barriers to acting, including a lack of timely feedback and possible information bias, influence their acceptance of telehealth services. Our research follows the HBM, which suggests the negative impact of HSE on telehealth usage, to propose the following hypothesis:

- Hypothesis 6: HSE is negatively associated with telehealth usage.

## Methods

### Data Source

This paper tests the hypotheses using data from cycle 6 of the HINTS, collected by the National Cancer Institute (NCI) [39]. The HINTS dataset has been widely adopted in health research to explore various dimensions and their impact on health communication, reflecting its significance in assessing patient perceptions in diverse health care contexts [40-42]. The cycle 6 survey was conducted between March and November 2022, and the final sample consists of 6252 respondents after removing outliers, missing values, and incorrectly answered questions. The survey uses a stratified random sampling approach to ensure adequate representation across demographic groups, including different age groups, education levels, geographic regions (rural vs urban), and racial backgrounds.

### Inclusion and Exclusion Criteria

We screened participants, including only those who had health visits at least once without counting an emergency room visit in the past 12 months. Our final dataset includes 5554 valid participants.

Among those respondents, 44.89% (2411 individuals) reported receiving telehealth services in the past 12 months. Among those who received telehealth services in the past 12 months, 44.01% (1061 individuals) were conducted via video, 34.47% (831 individuals) were conducted via phone call, and the remaining 21.53% (519 individuals) were conducted via a combination of video call and phone call. HINTS 6 also tracks whether the patients were offered the choice of telehealth services and why they chose telehealth or not. Among those patients provided with the opportunity for a telehealth visit, the top reason for not choosing it was preference for an in-person health service, while the top reason for choosing telehealth was its being recommended or required by the provider or perceived as more convenient than in-person care. These responses reveal that the patient’s choice is limited; they do not supply their own reasons for choosing telehealth, which calls attention to the issue that, besides the “have to” reason, insight into the factors that would encourage the use of telehealth from the patient’s perspective and self-motivated choice is needed.

Furthermore, the survey asked the primary reason for the most recent telehealth visit, and the responses are distributed evenly across annual checkups, minor illness, chronic disease management, and mental health concerns, with rare instances of telehealth visits for medical emergencies. Table 1 provides the respondents’ descriptive statistics. While the study sample is broadly representative of the US adult population, the slight overrepresentation of individuals with higher education levels and the underrepresentation of Hispanic participants may limit the generalizability of the findings to certain subgroups. However, the large sample size and inclusion of diverse demographic groups enhance the study’s ability to provide meaningful insights into telehealth usage across different populations.

**Table 1.** Respondents’ descriptive analysis.

Characteristics observations	Frequency (%)
Age (years)	
18‐34	786 (14.36)
35‐49	1058 (19.33)
50‐64	1588 (29.02)
65‐74	1247 (22.78)
75+	794 (14.51)
Education	
Less than high school	309 (5.95)
High school graduate	921 (17.73)
Some college	1491 (28.70)
Bachelor’s degree	1443 (27.78)
Postbaccalaureate degree	1031 (19.85)
Rural/urban	
High minority rural area	227 (4.09)
High minority urban area	3361 (60.51)
Low minority rural area	510 (9.18)
Low minority urban area	1456 (26.22)
Sex	
Male	1970 (37.94)
Female	3223 (62.06)
Race	
Non-Hispanic White	2935 (59.23)
Non-Hispanic Black or African American	797 (16.08)
Hispanic	826 (16.67)
Non-Hispanic Asian	236 (4.76)
Non-Hispanic Other	161 (3.25)

### Measurement and Variables

The primary dependent variable in this study is telehealth usage, measured using a single term derived from the following survey question: “In the past 12 months, did you receive care from a doctor or health professional using telehealth?” Participants who reported receiving telehealth services in the past 12 months were coded as “Yes,” while those who did not were coded as “No” [43].

Participants’ confidence in health information seeking was assessed by asking how confident they were in finding health information if needed. Responses were recorded on a 5-point Likert scale ranging from 1 (*Not confident at all*) to 5 (*Completely confident*). This measure aligns with prior research, which has used the same question to assess confidence in health information seeking [23,44].

PCC level was measured by assessing participants’ experiences with their health care provider in the past 12 months [20,28,44-46]. Multiple questions addressed various facets of communication; for example, “In the past 12 months, how often did your doctors, nurses, or other health professionals give you the chance to ask all the health-related questions you had?” Each item was rated on a 4-point Likert scale ranging from 1 (*Never*) to 4 (*Always*).

Health literacy barriers were assessed by administering a series of statements to participants addressing the challenges and concerns encountered when seeking health information [47,48], and each item was rated on a 4-point Likert scale ranging from 1 (*strongly agree*) to 4 (*strongly disagree*) to measure barriers. To mitigate any bias in implying negative judgment toward the clarity of health-related information, we reclassify the responses in this study and uniformly assign ascending numerical values (eg, 1 as *strongly disagree*, 4 as *strongly agree*). This variable was termed “health literacy barriers” to represent the obstacles inhibiting patients from obtaining, processing, and understanding basic health information and services [31].

Trust was measured using 3 items assessing participants’ confidence in cancer-related information from doctors, government health agencies, and scientists, based on a 4-point Likert scale (*Not at all*=1 to *A lot*=4). Although the HINTS dataset originally measured trust in cancer-related information, while our paper’s focus is on trust in information from a variety of sources, research suggests that trust in medical information sources generalizes across health domains due to the Halo Effect. The Halo Effect is a cognitive bias in which an individual’s trust in a particular person, institution, or concept extends to related but distinct areas [49,50]. In the context of telehealth, trust in health care providers may generalize to trust in telehealth platforms and digital health services. Similarly, trust in government health agencies and scientists regarding cancer information reflects broader trust in institutional health guidance, including digital health platforms and telehealth services. Thus, while our trust measures are framed in the context of cancer information, they serve as valid proxies for assessing general medical trust, which extends to telehealth adoption.

SDOH was measured through questions about food, housing, and transportation designed to evaluate whether patients faced challenges associated with SDOH. Responses were categorized as “Yes” (*sometimes true*, *often true*) and “No” (*never true*) [51].

HSE was measured using a single item assessing participants’ confidence in their ability to take good care of their health. Responses were recorded on a 5-point Likert scale ranging from 1 (*not at all confident*) to 5 (*completely confident*) [44].

SDOH, health literacy barriers, PCC, and Trust are measured using multiple respective questions, which collectively capture the shared variance among their related items. These multiscale variables are then analyzed to examine their impact on telehealth usage. Using different point-level Likert scales across multiple questions has also been approved as acceptable as long as it aligns with the research objectives and maintains clarity for respondents and no difference in predictive validity between multi-item and single-item measures [52].

Unlike our multiscale variables, confidence in health information seeking and HSE are measured using a single question, and the single-question approach can still effectively capture degrees of variation in the underlying variables [53,54], which demonstrates that single-item self-efficacy measures perform comparably to validated multi-item scales in predicting health-related behaviors. In the context of confidence in health information seeking, studies have used single-item measures to assess individuals’ confidence, aligning with our approach [23,44]. Given these findings, we determine that a single-item approach is appropriate for measuring constructs such as HSE and confidence in health information seeking.

Besides the main effect from the factors that interest us, prior research also suggests that telehealth usage varies by patient’s age, gender, ethnicity, and education level [55]; therefore, we include sociodemographics (age, race, gender, and education level) in our model as control variables. We also include participants’ community minority level to control the potential impact of technology familiarity and web development.

Table 2 provides the measurement items used with their constructs, corresponding references, and descriptive statistics—all variables’ Cronbach α ranges between 0.6 and 0.8 indicate strong internal consistency.

**Table 2.** Constructs, measures, and descriptive statistics.

Measurement item	Sample size, n (%)	Reference
Telehealth usage		[43]
In the past 12 months, did you receive care from a doctor or health professional using telehealth?		
Yes	2411 (44.89)	
No	2960 (55.11)	
Confidence in health information seeking		[23,44]
How confident are you that you can find helpful health resources on the web?		
Not confident at all	298 (5.47)	
A little confident	504 (9.26)	
Somewhat confident	2120 (38.94)	
Very confident	1888 (34.68)	
Completely confident	634 (11.65)	
Patient-centered communication (PCC)		[20,44-46]
In the past 12 months, how often did your doctors, nurses, or other health professionals give you the chance to ask all the health-related questions you had? (PCC1)		
Never	66 (1.21)	
Sometimes	509 (9.35)	
Usually	1693 (31.09)	
Always	3177 (58.35)	
In the past 12 months, how often did your doctors, nurses, or other health professionals explain things in a way you could understand? (PCC2)		
Never	63 (1.16)	
Sometimes	438 (8.06)	
Usually	1717 (31.59)	
Always	3217 (59.19)	
In the past 12 months, how often did your doctors, nurses, or other health professionals give the attention you needed to your feelings and emotions? (PCC3)		
Never	206 (3.80)	
Sometimes	952 (17.55)	
Usually	1871 (34.49)	
Always	2396 (44.17)	
In the past 12 months, how often did your doctors, nurses, or other health professionals help you deal with feelings of uncertainty about your health or health care? (PCC4)		
Never	299 (5.55)	
Sometimes	1053 (19.53)	
Usually	1855 (34.41)	
Always	2184 (40.51)	
In the past 12 months, how often did your doctors, nurses, or other health professionals involve you in decisions about your health care as much as you wanted? (PCC5)		
Never	119 (2.19)	
Sometimes	661 (12.18)	
Usually	1781 (32.82)	
Always	2865 (52.80)	
In the past 12 months, how often did your doctors, nurses, or other health professionals spend enough time with you? (PCC6)		
Never	215 (3.97)	
Sometimes	1040 (19.22)	
Usually	1876 (34.68)	
Always	2279 (42.13)	
In the past 12 months, how often did your doctors, nurses, or other health professionals make sure you understood the things you needed to do to take care of your health? (PCC7)		
Never	82 (1.51)	
Sometimes	536 (9.86)	
Usually	1703 (31.32)	
Always	3116 (57.31)	
Health literacy barriers		[47,48]
It took a lot of effort to get the information you needed (HLB1)		
Strongly disagree	686 (25.89)	
Somewhat disagree	974 (36.75)	
Somewhat agree	783 (29.55)	
Strongly agree	207 (7.81)	
You felt frustrated during your search for the information (HLB2)		
Strongly disagree	867 (32.94)	
Somewhat disagree	855 (32.48)	
Somewhat agree	711 (27.01)	
Strongly agree	199 (7.56)	
You were concerned about the quality of the information (HLB3)		
Strongly disagree	528 (20.02)	
Somewhat disagree	691 (26.19)	
Somewhat agree	986 (37.38)	
Strongly agree	433 (16.41)	
The information you found was hard to understand (HLB4)		
Strongly disagree	717 (27.26)	
Somewhat disagree	986 (37.49)	
Somewhat agree	751 (28.56)	
Strongly agree	176 (6.69)	
Trust		[20,56]
In general, how much would you trust information about cancer from a doctor? (Trust 1)		
Not at all	55 (1.00)	
A little	201 (3.67)	
Some	1157 (21.13)	
A lot	4062 (74.19)	
In general, how much would you trust information about cancer from government health agencies? (Trust 2)		
Not at all	430 (8.07)	
A little	975 (18.30)	
Some	2382 (44.71)	
A lot	1541 (28.92)	
In general how much would you trust information about cancer from scientists? (Trust 3)		
Not at all	244 (4.54)	
A little	517 (9.63)	
Some	1550 (28.86)	
A lot	3060 (56.97)	
Social determinants of health (SDOH) (food, housing, and transportation)		[51,57]
Someone in your household cut the size of meals or skipped meals because there wasn’t enough money for food (SDOH1)		
Often true/sometimes true	665 (12.55)	
Never true	4635 (87.45)	
Someone in your household was not able to afford to eat balanced meals (SDOH2)		
Often true/sometimes true	782 (14.79)	
Never true	4505 (85.21)	
Someone in your household was worried about being forced to move (eg, because of eviction or foreclosure) (SDOH3)		
Often true/sometimes true	520 (9.87)	
Never true	4747 (90.13)	
Lack of reliable transportation kept someone in your household from medical appointments, work, or from getting things needed for daily living (SDOH4)		
Often true/sometimes true	604 (11.43)	
Never true	4679 (88.57)	
Health self-efficacy		[44,58,59]
Overall, how confident are you about your ability to take good care of your health?		
Not confident at all	65 (1.21)	
A little confident	227 (4.23)	
Somewhat confident	1238 (23.09)	
Very confident	2391 (44.60)	
Completely confident	1440 (26.86)	

### Ethical Considerations

The original HINTS 6 data collection by the NCI received approval from the Westat institutional review board (IRB), and all participants provided informed consent prior to participation. The dataset is fully deidentified, ensuring that no personally identifiable information is available. Our study involved secondary analysis of this publicly available, deidentified dataset. In accordance with US federal regulations (45 CFR 46) and institutional policies, secondary analyses of deidentified, publicly available data do not require additional IRB review. We adhered to ethical standards outlined in the WMA Declaration of Helsinki and the Belmont Report. No compensation was provided to participants, as the original data collection handled this aspect. All findings are reported in a manner that maintains participant confidentiality and adheres to ethical standards of data use. Further details on the survey instrument, and the methodology and data handling for HINTS 6, can be explored through the NCI’s official resources [60].

### Statistical Analysis

We analyze our survey data using SmartPLS 3.0, using partial least squares (PLS). Previous studies have argued that PLS has advantages over covariance-based structural equation modeling when the research question is focused more on the nature of the relationship rather than the magnitude of that relationship [61]. SmartPLS 3.0 also allows us to test the quadratic effect related to H1. Following prior research using the PLS model, we tested our model using a 2-step approach [62,63]. We addressed the reliability and validity of the measurement model in the first step and tested the structural model in the second step.

### Measurement Model

We ensure convergent validity by verifying three criteria: (1) all item loadings have composite reliability above 0.60 and are statistically significant [64], (2) the composite reliability of all constructs exceeds 0.70 [65], and (3) the average variance extracted is greater than 0.5 [65]. Table 3 shows the item loadings and cross-loadings, and Table 4 provides the construct validity matrix. Additionally, we check for multicollinearity using the variance inflation factor. All variance inflation factor scores range between 1.00 and 3.44, well below the recommended threshold value of 5, indicating no multicollinearity issues [66].

**Table 3.** Item loadings and cross-loadings.

	Telehealth usage	InfoSeeking^a^	PCC^b^	HLB^c^	Trust	SDOH^d^	HSE^e^
Telehealthusage	1	0.078	−0.019	0.017	0.061	0.073	−0.038
InfoSeeking	0.078	1	0.129	−0.217	0.270	−0.056	0.182
PCC1	−0.025	0.108	0.843	−0.125	0.168	−0.171	0.243
PCC2	0.007	0.135	0.855	−0.145	0.195	−0.163	0.249
PCC3	−0.060	0.098	0.833	−0.107	0.117	−0.147	0.246
PCC4	−0.031	0.092	0.827	−0.128	0.128	−0.150	0.245
PCC5	−0.017	0.113	0.849	−0.114	0.173	−0.172	0.256
PCC6	−0.019	0.080	0.839	−0.134	0.125	−0.170	0.238
PCC7	-0.013	0.120	0.886	-0.139	0.183	-0.159	0.265
HLB1	0.011	−0.184	−0.122	0.869	−0.121	0.107	−0.091
HLB2	0.013	−0.195	−0.134	0.877	−0.109	0.095	−0.103
HLB3	0.024	−0.170	−0.111	0.780	−0.102	0.084	−0.060
HLB4	0.009	−0.176	−0.138	0.812	−0.155	0.073	−0.155
Trust1	0.028	0.148	−0.104	−0.123	0.725	−0.118	0.111
Trust2	0.052	0.248	0.103	−0.113	0.797	−0.082	0.046
Trust3	0.060	0.230	0.084	−0.077	0.807	−0.063	0.053
SDOH1	0.048	−0.049	−0.140	0.076	−0.100	0.820	−0.165
SDOH2	0.060	−0.045	−0.154	0.107	−0.095	0.855	−0.199
SDOH3	0.067	−0.025	−0.147	0.078	−0.075	0.762	−0.176
SDOH4	0.053	−0.060	−0.165	0.075	−0.087	0.722	−0.223
HSE	−0.038	0.182	0.294	−0.112	0.089	−0.241	1

a InfoSeeking: confidence in health information seeking.

b PCC: patient-centered communication.

c HLB: health literacy barriers.

d SDOH: social determinants of health.

e HSE: health self-efficacy.

**Table 4.** Convergent validity, discriminant validity, and correlation of constructs.

Construct	CA^a^	CR^b^	AVE^c^	Telehealthusage	InfoSeeking	PCC^d^	HLB^e^	Trust	SDOH^f^	HSE^g^
Telehealthusage	1	1	1	1	N/A^h^	N/A	N/A	N/A	N/A	N/A
InfoSeeking	1	1	1	0.078	1	N/A	N/A	N/A	N/A	N/A
PCC	0.935	0.947	0.719	0.024	0.130	1	N/A	N/A	N/A	N/A
HLB	0.855	0.902	0.698	0.018	0.235	1	1	N/A	N/A	N/A
Trust	0.671	0.821	0.604	0.073	0.328	0.234	0.253	1	N/A	N/A
SDOH	0.800	0.870	0.626	0.081	0.064	0.220	0.168	0.155	1	N/A
HSE	1	1	1	0.038	0.182	0.303	0.120	0.110	0.270	1

a CA: Cronbach α.

b CR: composite reliability.

c AVE: average variance extracted.

d PCC: patient-centered communication.

e HLB: health literacy barriers.

f SDOH: social determinants of health.

g HSE: health self-efficacy.

h N/A: not applicable.

## Results

### Common Method Variance

Common method variance may exist if independent and dependent variables are measured in the same survey [41,67]. To address common method variance, we use multiple methods. First, we conduct Harman’s 1-factor analysis [68] and find that the largest single component accounts for only 21.2%, well below the threshold value of 50%. Second, we use the marker approach used by Lindell and Whitney [69]. Specifically, we use “ElectCigLessHarm” (measuring levels at which participants think e-cigarettes contain more or less nicotine than regular cigarettes) as a marker variable theoretically unrelated to telehealth usage.

Table 5 shows the correlation between the principal variables and the marker variable. The correlation between the marker variable and the other principal variables is low and mostly insignificant, and all coefficients meet the threshold value of 0.1 [69]. However, the correlation between HSE and the marker variable is statistically significant (β=.051; *P*<.01). While this correlation is weak, it suggests a potential, albeit minor, influence of common method variance on the measurement of HSE. To assess the impact of this correlation on the main analysis, we compare the results of the model with and without the marker variable. The direction and significance levels of the hypothesized paths remain unchanged, indicating that the observed correlation does not substantially affect the results. These analyses provide strong evidence that common method variance is not a significant concern in this study, thereby strengthening the validity of our findings.

**Table 5.** Correlation with marker variable.

	Telehealth usage	InfoSeeking	PCC^a^	HLB^b^	Trust	SDOH^c^	HSE^d^
With marker variable “ElectCigLessHarm”							
Correlation	0.002	−0.047	0.033	−0.015	−0.063	0.007	0.051
*P* value	.77	.15	.17	.45	<.10	.35	<.01

a PCC: patient-centered communication.

b HLB: health literacy barriers.

c SDOH: social determinants of health.

d HSE: health self-efficacy.

### Structural Model

**Figure 2.**
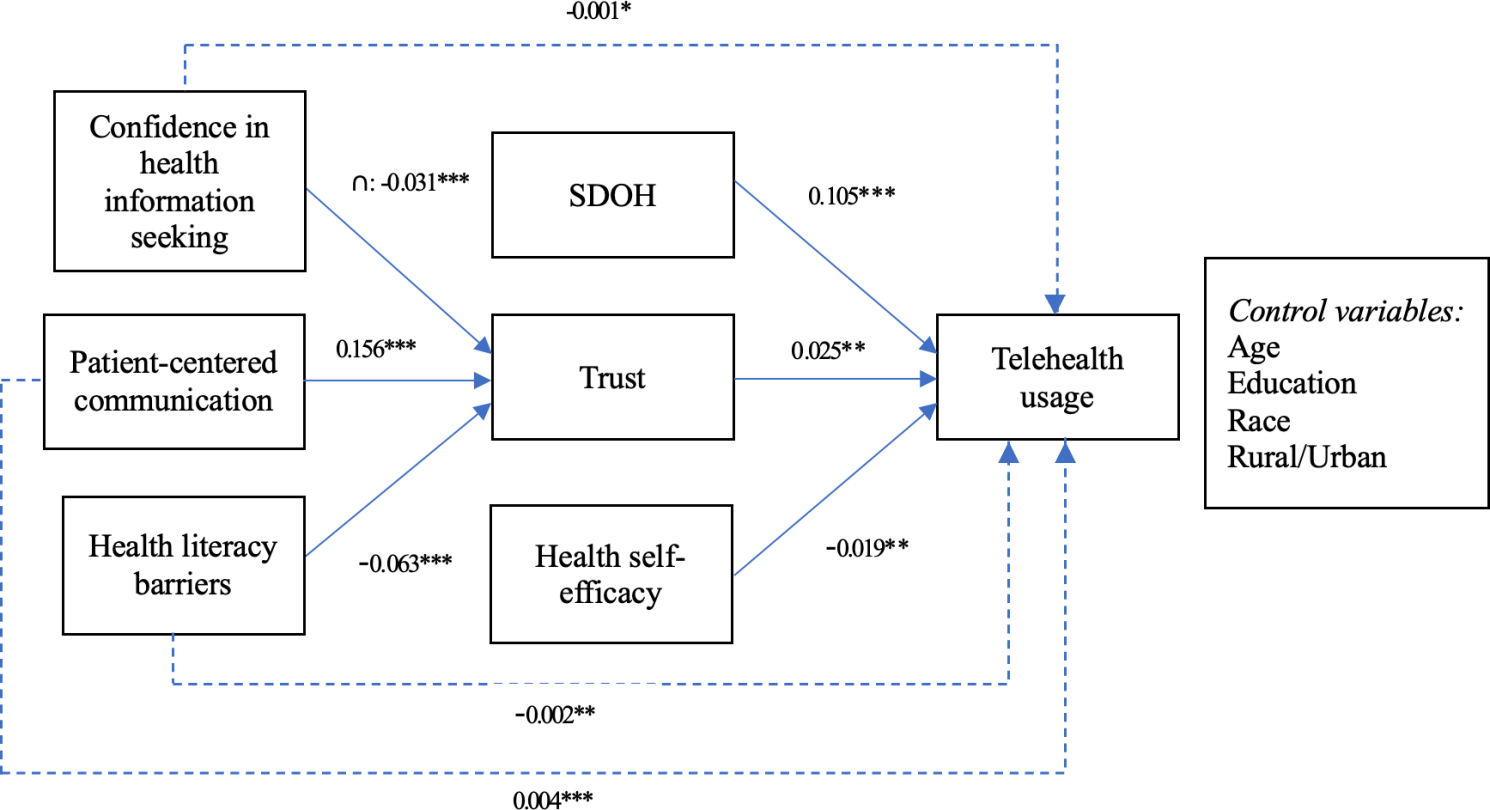
Structural model notes. *P<.05, **P<.01, ***P<.001. ∩: quadratic item.

The results of our structural analysis, as shown in Table 6 and Figure 2, support all 6 hypotheses proposed in our study. Our findings reveal a curvilinear relationship between confidence in health information seeking and trust, with a negative association (β=−.031; *P*=.002) supporting hypothesis 1. The effects of PCC (β=.156 *P*<.001) and health literacy barriers (β=−.063; *P*<.001) on trust are significant and thus support hypothesis 2 and hypothesis 3, respectively. Trust positively and significantly impacts telehealth usage (β=.025; *P*.001), thereby supporting hypothesis 4. Finally, the impact from SDOH is positively associated with telehealth usage (β=.105; *P*<.001), while HSE is negatively associated with telehealth usage (β=−.019; *P*=.007), thus supporting hypothesis 5 and hypothesis 6, respectively. Regarding the control variables, our analysis aligns with previous research from Williams and Shang [55], indicating significant effects of age and education level on telehealth usage. Younger patients with higher education levels are more likely to use telehealth services. Also, compared with males, females are more willing to use telehealth [70]. However, race or ethnicity and community minority level do not significantly influence telehealth usage in our analysis.

**Table 6.** Results of structural equation modeling.

Hypothesis	Independent variable	DV^a^	Predicted relationship	β	*P* value	Supported/not supported
H1^b^	InfoSeeking	Trust	∩^c^	−.031	.002	Supported
H2	Patient-centered communication	Trust	+	.156	<.001	Supported
H3	Health literacy barriers	Trust	−	−.063	<.001	Supported
H4	Trust	Telehealth usage	+	.025	<.001	Supported
H5	SDOH^d^	Telehealth usage	+	.105	<.001	Supported
H6	Health self-efficacy	Telehealth usage	−	−.019	.007	Supported

a DV: dependent variable.

b H: hypothesis.

c Quadratic item.

d SDOH: social determinants of health.

These findings provide valuable insights into the factors influencing trust and telehealth usage, highlighting the importance of patient engagement, health literacy, and SDOH in shaping health care behaviors and promoting telehealth usage. Table 7 shows the mediation analysis results for telehealth usage with confidence in health information seeking, PCC, and health literacy barriers, showing that all indirect impacts are significant.

**Table 7.** Mediation results of structural equation modeling.

Independent variable	Mediations	Dependent variable	β	*P* value	Significant
Confidence in health information seeking	Trust	Telehealth usage	−.001	.021	Yes
Patient-centered communication	Trust	Telehealth usage	.004	.000	Yes
Health literacy barriers	Trust	Telehealth usage	−.002	.003	Yes

## Discussion

### Key Findings

Telehealth has emerged as a transformative approach to health care delivery, offering remote access to medical services through technology. This study examines the factors influencing telehealth usage, focusing on patient characteristics such as confidence in health information seeking, PCC, health literacy barriers, trust, SDOH, and HSE. Our findings reveal that trust, SDOH, and HSE significantly influence telehealth usage, with trust playing a mediating role in the relationship between PCC, health literacy barriers, and telehealth adoption.

We observe an inverted U-shaped relationship between confidence in health information seeking and trust, suggesting that moderate levels of confidence enhance trust, while excessive confidence may lead to skepticism or information overload. This finding aligns with prior research on the dual role of information-seeking behavior in shaping trust and engagement in health care services [22]. Moderate levels of information search competence may promote trust by enhancing knowledge acquisition, and such trust supports greater telehealth usage.

Our study also highlights the critical role of PCC in fostering trust and engagement in telehealth. Patients who experience higher levels of PCC report greater trust in health care providers and are more likely to use telehealth services. Conversely, health literacy barriers negatively impact trust and telehealth usage, underscoring the need for interventions to improve health literacy to promote telehealth usage [24].

Interestingly, we find that individuals facing SDOH challenges, particularly food insecurity, are more likely to use telehealth services. This suggests that telehealth can mitigate barriers related to socioeconomic disparities, offering a viable solution for improving health care access among disadvantaged populations [71]. However, the weaker associations for transportation and housing insecurity indicate that telehealth alone may not fully address all SDOH-related challenges, necessitating targeted interventions to complement telehealth services.

Finally, our research reveals a negative association between HSE and telehealth usage, contrasting with prior research. This finding suggests that individuals with high HSE, who are more confident in managing their health, may prefer traditional in-person consultations over telehealth. This highlights the need for tailored telehealth strategies that accommodate the preferences of individuals with varying levels of HSE.

### Practical Implications

The insights gained from our study hold significant practical implications for health care providers, policy makers, and stakeholders involved in health care systems and telehealth implementation. First, we observe an inverted U-shaped association between patients’ confidence in health information seeking and trust. This indicates that while moderate levels of confidence in health information seeking enhance trust, excessively high levels may lead to skepticism or mistrust. Health care providers should therefore tailor communication and educational strategies to match the information-seeking confidence of different patient groups, which can strengthen patient trust and engagement. For instance, for individuals with low confidence, offering additional guidance and support can enhance trust and engagement. For those with high confidence, providing detailed, evidence-based information can prevent skepticism and foster trust.

Second, health care providers should prioritize implementing a PCC environment to foster trust and engagement among telehealth users, such as active listening, empathy, and shared decision-making, to create a supportive telehealth environment. This can strengthen patient-provider relationships and increase telehealth adoption.

Third, enhancing health literacy is crucial for building trust and increasing telehealth usage among diverse patient populations. Health care organizations can introduce interventions such as patient navigation programs, medical language translation, and health literacy assessments integrated into telehealth platforms to ensure that all users can access and comprehend health information.

In addition, policy makers should work to address SDOH to maximize telehealth’s potential, particularly for underserved populations. This could involve expanding telehealth access in rural and low-income areas, offering linguistically and culturally appropriate telehealth services, and addressing digital disparities by improving infrastructure. Furthermore, telehealth adoption strategies should consider tailoring interventions to accommodate individuals with high HSE, potentially integrating elements that address their preference for face-to-face interactions to optimize health care delivery. Offering hybrid models of care that combine telehealth with traditional health care services can optimize patient satisfaction and engagement.

### Limitations and Future Research

This study has several limitations. First, our use of the HINTS dataset restricts the range of variables available for analysis, particularly regarding trust, which was measured in the context of cancer-related information. Future research could benefit from using more comprehensive and diverse datasets that capture a wider range of variables relevant to telehealth usage, including patients’ health conditions and disease types. Second, the cross-sectional design of the study limits our ability to establish causal relationships between the variables. Longitudinal or experimental studies are needed to explore the dynamic changes in telehealth usage over time and the causal pathways underlying the observed relationships.

Finally, our study primarily focuses on individual-level factors. Future research should examine contextual and organizational factors, such as health care system characteristics, policies, and environmental influences, to provide a more comprehensive understanding of the determinants driving telehealth adoption.

### Conclusions

This study provides critical insights into the factors influencing telehealth usage, highlighting the interplay between patient characteristics, trust, and health care access. By integrating perspectives from the TAM, HBM, and SEM, our research offers a holistic framework for understanding telehealth adoption.

The findings underscore the importance of fostering trust through PCC, addressing health literacy barriers, and leveraging telehealth to mitigate socioeconomic disparities. Tailored interventions that consider the diverse needs and preferences of patients can enhance telehealth usage and improve health care outcomes.

Looking forward, this study lays the groundwork for future research aimed at optimizing telehealth strategies and advancing equitable access to digital health care. By continuing to investigate the evolving dynamics of telehealth adoption, we can better leverage technology to address health care disparities and improve public health outcomes.

## Abbreviations

HBM: Health Belief Model
HINTS: Health Information National Trends Survey
HSE: health self-efficacy
IRB: institutional review board
NCI: National Cancer Institute
PCC: patient-centered communication
PLS: partial least squares
SDOH: social determinants of health
SEM: Social Ecological Model
TAM: Technology Acceptance Model
